# Identifying, Quantifying, and Recovering a Sorbitol-Type Petrochemical Additive in Industrial Wastewater and Its Subsequent Application in a Polymeric Matrix as a Nucleating Agent

**DOI:** 10.3390/molecules28134948

**Published:** 2023-06-23

**Authors:** Joaquín Hernández-Fernández, Esneyder Puello-Polo, Edgar Marquez

**Affiliations:** 1 Chemistry Program, Department of Natural and Exact Sciences, San Pablo Campus, University of Cartagena, Cartagena 130015, Colombia; 2 Chemical Engineering Program, School of Engineering, Universidad Tecnológica de Bolivar, Parque Industrial y Tecnológico Carlos Vélez Pombo Km 1, Vía Turbaco, Cartagena 130001, Colombia; 3 Department of Natural and Exact Science, Universidad de la Costa, Barranquilla 080002, Colombia; 4 Group de Investigación en Oxi/Hidrotratamiento Catalítico y Nuevos Materiales, Programa de Química-Ciencias Básicas, Universidad del Atlántico, Puerto Colombia 081001, Colombia; esneyderpuello@mail.uniatlantico.edu.co; 5 Grupo de Investigaciones en Química y Biología, Departamento de Química y Biología, Facultad de Ciencias Básicas, Universidad del Norte, Carrera 51B, Km 5, Vía Puerto Colombia, Barranquilla 081007, Colombia

**Keywords:** polypropylene, clarifying agent, Millad NX 8000, solid phase extraction, purity, recovery, crystallization, degradation

## Abstract

Currently, polypropylene (PP) is highlighted using sorbitol-based clarifying agents since these agents are high quality, low cost, and work as a barrier against moisture, which makes PP ideal for packaging food, beverages, and medical products, among others. The use of analytical methods capable of recovering these additives in wastewater streams and then reusing them in the PP clarification stage represents an innovative methodology that makes a substantial contribution to the circular economy of the PP production industry. In this study, a method of extraction and recovery of the Millad NX 8000 was developed. The additive was recovered using GC-MS and extracted with an activated carbon column plus glass fiber, using an injection molded sample, obtaining a recovery rate greater than 96%. TGA, DSC, and FTIR were used to evaluate the recovered additive’s glass transitions and purity. The thermal degradation of the recovered additive was found to be between 340 and 420 °C, with a melting temperature of 246 °C, adopting the same behavior as the pure additive. In FTIR, the characteristic absorption peak of Millad NX 8000 was observed at 1073 cm^−1^, which indicates the purity of the extracted compound. Therefore, this work develops a new additive recovery methodology with high purity to regulate the crystallization behavior and of PP.

## 1. Introduction

**Figure 1:**
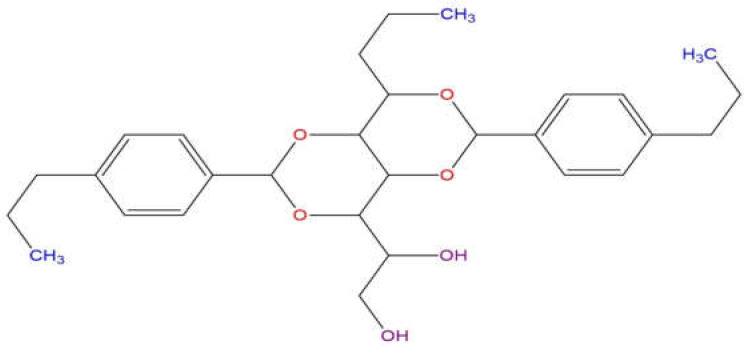
Chemical structure of Millad NX 8000.

Today, environmental conservation has emerged as a crucial concern for the sustainable progress of our society, driven by the increasing recognition of the detrimental consequences posed by our industrial, economic, and societal practices on the environment and the overall quality of life [1]. One of the most critical challenges we face is properly managing wastewater since its untreated discharge can have significant negative consequences for aquatic ecosystems and human health [2,3,4,5]. One of the main parties responsible for the emission of a large number of pollutants into water is the petrochemical industry since they unintentionally or deliberately release chemical substances in the production of polymers [6,7,8]. Within the broad spectrum of pollutants present in wastewater, chemical additives play a prominent role due to their wide use in various industries [7]. The additive 1,2,3-trideoxy-4,6:5,7-bis-*O*-[(4-propylphenyl)methylene]nonitol is one of the substances present in wastewater, known commercially as Millad NX 8000 (Figure 1), a substance widely used in the manufacture of plastic products, especially in the production of bottles and containers, which are present in many aspects of daily life and their production has increased dramatically in the last 50 years [9]. Millad NX 8000 belongs to a class of additives known as nucleants, which are used to improve polymers’ mechanical and optical properties. Millad NX 8000 is used from 0.01% to 1% during polypropylene (PP) manufacturing to improve its transparency and resistance to high temperatures [10]. Since polypropylene crystallizes very slowly by forming relatively large spherulites [11], this causes considerable light scattering and opacity in the polymer [11]. During the cooling process, the segregation and crystallization of the sorbitol derivatives result in the formation of spherulites, which are observed as fibers with a diameter of 10 nm [12,13,14,15,16]. Millad acts as a nucleation and clarification agent, allowing the spherulites formed to be smaller than the wavelength of light, and therefore a polymer with less opacity.

In the last 39 years, various types of nucleating agents with a clarifying effect on PP have been developed. One main disadvantage of PP clarifiers has been their organoleptic problems (taste and odor) since most of these clarifiers are based on sorbitol acetal compounds, which hydrolyze during the PP processing stage, leading to the release of free aldehydes, which then cause unpleasant flavors and odors in PP [17]. Among its most recent products and with innovative chemistry is the Millad NX 8000, which is characterized by providing a decrease in turbidity in the PP greater than 50% concerning previous clarifying agents; it is also free of the odors and flavors that other nucleating agents used in the packaging products made of PP transmits [17]. The national and international industries that produce PP use billions of tons of these additives per year, and it is known that their production processes are not entirely efficient. Significant amounts of these additives can end up in wastewater. This can have negative consequences for the environment and human health since it is known that many of these chemical additives can be toxic and persistent in the environment [17], for which Millad NX 8000 can be considered a persistent organic pollutant (POP). Considering that this additive is frequently used in the production of plastic products and different studies have been carried out for its detection in macro and microplastic waste, it is to be expected that they are found in environmental matrices (water, sediments, and biota) and may represent an ecological concern [18]. The existence of additives, such as Millad NX 8000, in polymer matrices is potentially a severe factor in the physical recycling of plastics, which delays progress toward the circular economy, which is where the plastics industry intends to go [19].

In a study carried out in rats for 90 days to observe the acute toxicity, sensitization, irritation, oral toxicity, and possible effects on the public and environmental health of the additive studied in this article, it was found that Millad NX 8000, not being chemically bound to the polymeric matrix, has a high potential to migrate from the articles in which it is incorporated, which allows the public to be exposed to this chemical [20]. Some histopathological changes were observed in rats exposed to high doses (1682 mg/kg) [20]. The toxicity data for this additive report a low potential to cause adverse effects after dermal or oral exposure. However, not enough studies have been carried out to quantitatively evaluate the risks of its use in materials in contact with food [20]. Millad NX 8000 is slightly irritating to the eyes and may cause respiratory tract irritation, but acute and long-term effects on the lungs/respiratory system have not been thoroughly studied. The presence of Millad NX 8000 in wastewater poses environmental and health problems due to its chemical properties and potential impact on aquatic ecosystems. Therefore, it is necessary and urgent to recover this additive from wastewater to minimize the associated risks and promote sustainable practices in waste management. The scarce information related to the environmental impact of the Millad in different bodies of water is also associated with the limitations presented in the identification and quantification processes in the said bodies of water [21]. This is most likely due to the poor solubility of these substances in various solvents, which complicates the preparation of stock solutions and the choice of a suitable extraction solvent; unfortunately, there are minimal data available on the solubility and thermal properties of such a substance [22,23,24].

Among the various instrumental techniques employed to investigate Millad NX 8000 and its role as a nucleation/crystallization agent in polymers, Fourier transform infrared spectroscopy (FTIR) [25,26] and scanning electron microscopy (SEM) [26] have been utilized. Noteworthy studies, such as the comprehensive research conducted by Smith et al. on the binary system of polypropylene and 1,3:2,4-bis(3,4-dimethylbenzylidene) sorbitol (DMDBS), have successfully determined the solubility of the additive using optical microscopy (OM) and differential scanning calorimetry (DSC). Additionally, they have developed a non-equilibrium phase diagram that intricately describes the various phases formed during cooling or heating, and their structures across the entire composition and within a broad temperature range [27]. In another study, gas chromatography-mass spectrometry (GC/MS) techniques coupled with microwave-assisted extraction were employed to identify the nucleating agents in plastic materials [28].

Presently, it is evident that a comprehensive and reliable analytical methodology for the extraction and quantification of Millad NX 8000, a common component found in industrial waste, has not been established. Consequently, the production process of polypropylene (PP) is not environmentally sustainable. Therefore, there is a critical necessity to develop robust analytical methods that can accurately identify, quantify, and recover Millad NX 8000, addressing the urgent need for environmentally friendly practices in the PP manufacturing industry.

The objective of this research is to propose a suitable analytical methodology for the extraction and recovery of Millad NX 8000 present in wastewater using solid-phase extraction with an adsorbent phase of glass fiber and activated carbon. GC-MS technique was used for quantification and FTIR, DSC, TGA and UV-VIS were used to characterize the recovered material. The recovered additive was added to a PP matrix to evaluate its performance via FTIR, DSC and TGA.

In doing so, this study makes a contribution to the circular economy of petrochemical plants producing PP worldwide, providing new knowledge that contributes to better monitoring and optimization of all industrial processes in which this additive is used.

## 2. Results and Discussion

### 2.1. Identification, Quantification, Repeatability, Reproducibility, and Linearity Analysis of Multiple Millad NX 8000 Standards

The analysis of Millad NX 8000 plays a crucial role in determining the success of the additive recovery process, as it provides essential insights into the feasibility of the recovery. The obtained results will serve as a key indicator of the effectiveness of the recovery process. Therefore, it is imperative to carry out the analysis meticulously and promptly to ensure accurate and reliable findings.

#### 2.1.1. Repeatability, Reproducibility, and Linearity of the Extraction of the Millad NX 8000 with a Column of Activated Carbon Plus Glass Fiber with DCM

**Figure 2:**
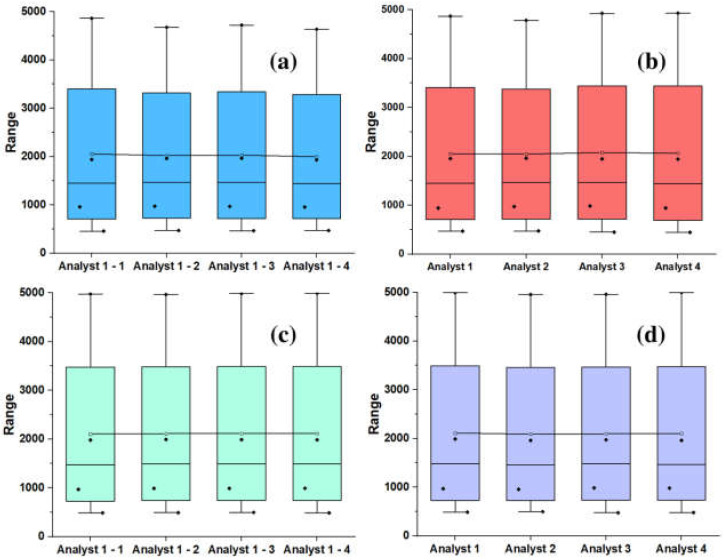
(**a**) Repeatability of recovery and extraction in whisker box plots; (**b**) Reproducibility of recovery and extraction in whisker box plots; (**c**) Repeatability of data obtained using GC-MS plotted in whisker boxes; (**d**) Reproducibility of data obtained via GC-MS plotted in whisker boxes.

Five tests were carried out for each sample via the GC/MS technique using the same operator, standard, and five concentrations between 0 and 5000 ppm to determine the repeatability (RPED) of the process. Activated carbon and fiberglass column was used to separate and analyze the recovered additive. The activated carbon is used as an absorbent to retain the organic compound. At the same time, the fiberglass supports the column and helps maintain the porous structure of the activated carbon. Dichloromethane (DCM) was used as the carrier gas because it has low polarity and high dissolving capacity for volatile organic compounds. According to the average values obtained, which must be less than 20% for the day, the method’s precision can be validated with certainty. The bibliography recommends that it is acceptable for the deviation to be less than 15% of the predicted value [29,30]. Table 1 shows the precision values of the data obtained in the additive extraction stage. Table 2 shows these values analyzed via ANOVA using the Tukey method with a 95% confidence interval of (α) 0.05. The analysis revealed that all means are associated with the same label (A), with a *p*-value of 1, indicating that the means do not share significant differences. With this, it can be stated with 95% confidence that the analysis is replicable. Figure 2 shows the distribution of repeatability and reproducibility data (RPOD) graphically. The same RPED validation standards were used for the RPOD.

**Figure 3:**
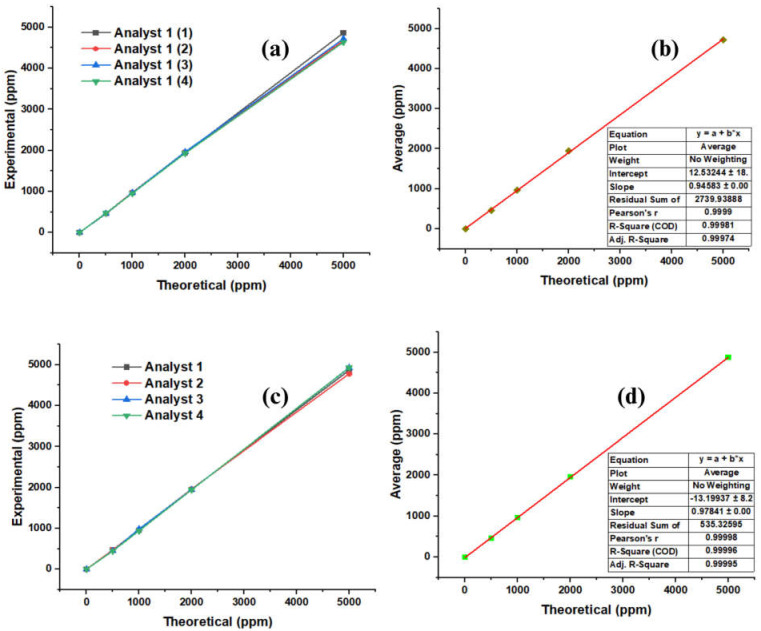
Linearity of the repeatability and reproducibility of the extraction method. (**a**) Repeatability of the standard; (**b**) average repeatability of the standard; (**c**) reproducibility of the standard; (**d**) average reproducibility of the standard.

Figure 3a shows the behavior of the standards. Initially, at concentrations of 0, 500, 1000, and 2000 ppm, it is observed that the sample follows a linear behavior. However, the precision decreases precisely at the concentration of 5000 ppm analyzed via analyst 1 in the first run. Figure 3b shows the average net recovery values recorded by analyst one compared to the theoretical concentrations, where the linear correspondence between the values can be seen, which highlights that the methodology used for the extraction of the additive in this study has a high extraction and recovery performance since the linear regression performed for this data set shows an R^2^ of 0.99981 with a correlation coefficient of 0.9999. Figure 3c graphically shows the values obtained for the RPOD, following the same analysis method for the RPED. Here, it is observed how the standards follow the same behavior as in Figure 3a, except that the change in the concentration of 5000 ppm occurred with analyst 2; however, when plotting the average values against the theoretical concentrations, an R^2^ of 0.9996 was obtained with a correlation coefficient of 0.9998, confirming that the extraction of Millad NX 8000 can indeed be achieved with a high degree of purity using this method, regardless of the analyst.

#### 2.1.2. Repeatability, Linearity, and Reproducibility of Millad NX 8000 via GC-MS

In addition to using GC-MS for the recovery of the additive, thanks to its high sensitivity and ability to separate and detect our sample, this was also used to identify the recovered additive, detect the different components of said sample and quantify its concentration. For the repeatability and reproducibility of the method via GC-MS, the same methodology of Section 2.1.1 was carried out. Table 3 shows the values of the data obtained experimentally for the quantification of the additive. Low RSD values were obtained within the acceptable range (below 15%), where the maximum reported value was 0.9 for concentrations of 500 and 1000 ppm, with an error of 2.0 and 1.5, respectively. The ANOVA analysis carried out for the GC-MS of Millad NX 8000 (recovered) is found in Table 2, where it can be seen that the means do not present significant differences since the groups of these were located in the same literal (A), with a *p*-value of 1.00.

**Figure 4:**
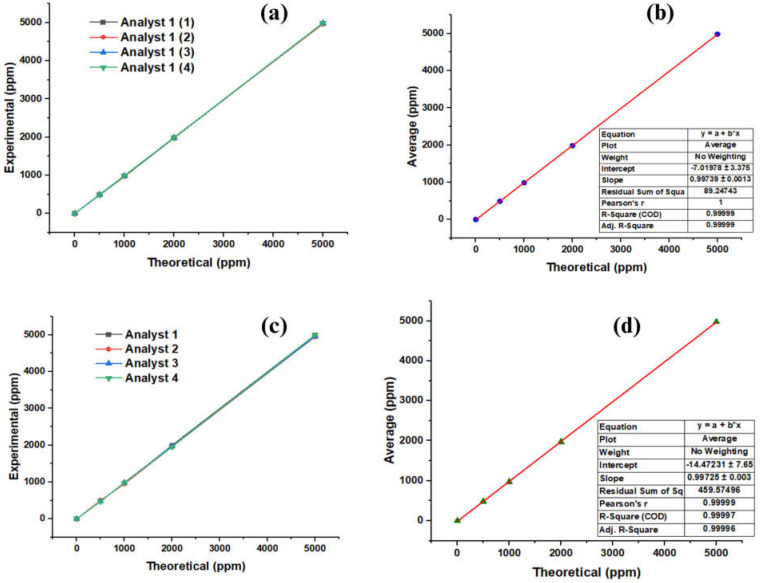
Linearity of repeatability and reproducibility measurements for quantification via GC-MS. (**a**) Repeatability of the standard; (**b**) average repeatability of the standard; (**c**) reproducibility of the standard; (**d**) average reproducibility of the standard.

The theoretical concentrations compared to those obtained experimentally by each analyst, determined via GC-MS, are found graphically in Figure 4. Figure 4a shows that each test adopts a linear behavior, which allows us to verify that the method efficiently quantifies the additive. Figure 4b shows the relationship between the theoretical data of the concentrations and the experimental ones, reporting an R^2^ value of 0.99999 with a correlation coefficient of 0.99999. These data lead us to infer with all certainty that the model can favorably quantify the concentrations.

### 2.2. Reproducibility and Repeatability Analysis of the Extraction of Industrial Wastewater Samples

#### 2.2.1. Reproducibility Analysis of the Industrial Wastewater Samples

The samples were 30 in total, taken on 30 consecutive days and analyzed via four different analysts on the same days. RSD and standard deviations of the samples were obtained, where the highest value was 5.9%, with a typical error of 42%. The highest recovery was 97.83%, with a standard error of 8% and an RSD of 2.2%. The highest concentration value reported in the samples was on day 1, with 4257 ppm, and the lowest was found on day 29, with a value of 275 ppm, as shown in Table 4.

#### 2.2.2. Linearity and Distribution of Industrial Wastewater Sample Data

**Figure 5:**
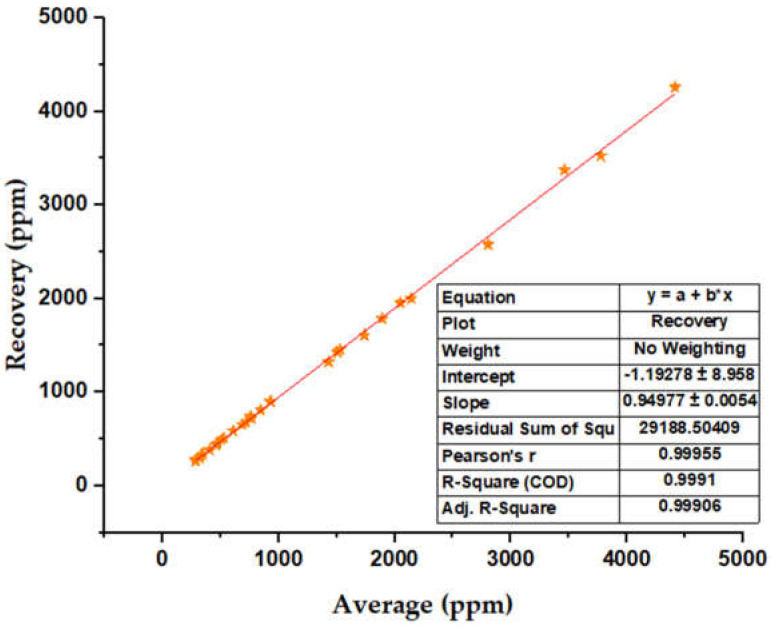
Analyte recovery based on mean sample concentrations.

Figure 5 shows the average values obtained by each analyst against the net recovery (ppm) in ascending order. The linear correspondence between the average values and the net recovery is estimated, which emphasizes and validates that the methodology used for the recovery of the additive of interest does have a high recovery performance since the linear regression carried out for this data set shows an (R^2^) of 0.9991 and a correlation coefficient of 0.99955.

### 2.3. Millad NX 8000 Peak Wavelength Measurement

**Figure 6:**
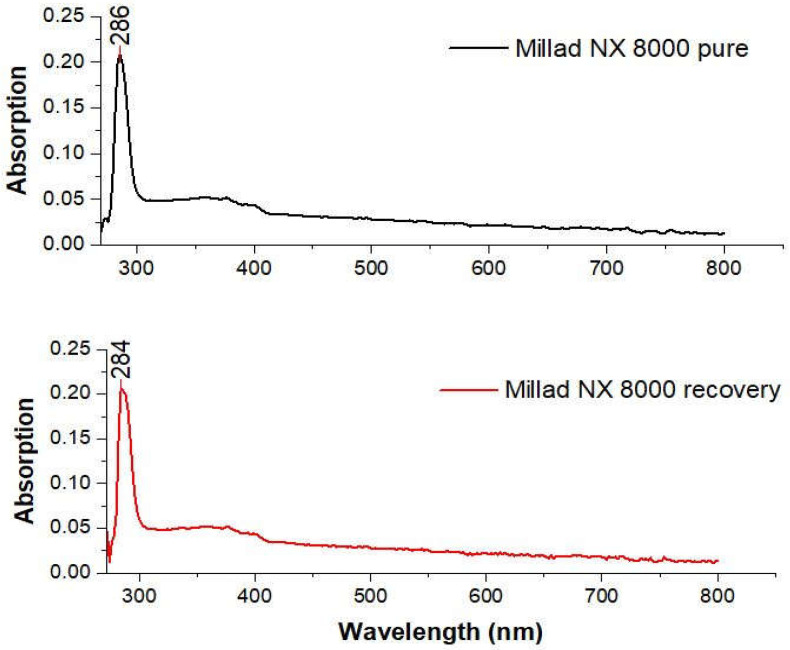
Lamda Máximo de Millad NX 8000 pure and recovery.

Scanning was carried out in the region of the UV-vis spectrum between 190 and 800 nm. However, the initial solution with which the scan was attempted was 100 ppm. At this concentration, the scan presented a low resolution, given that the molar absorptivity of the Millad at said concentration was too high, behaving like a solvent and evading Beer’s law. The scan was repeated with lower concentrations until reaching 50 ppm, where the best resolution was found. The λ_max_ of the NX 8000 Millad standard was determined at 286 nm (Figure 6).

#### Linearity Test of the UV-Vis Spectrophotometric Method

**Figure 7:**
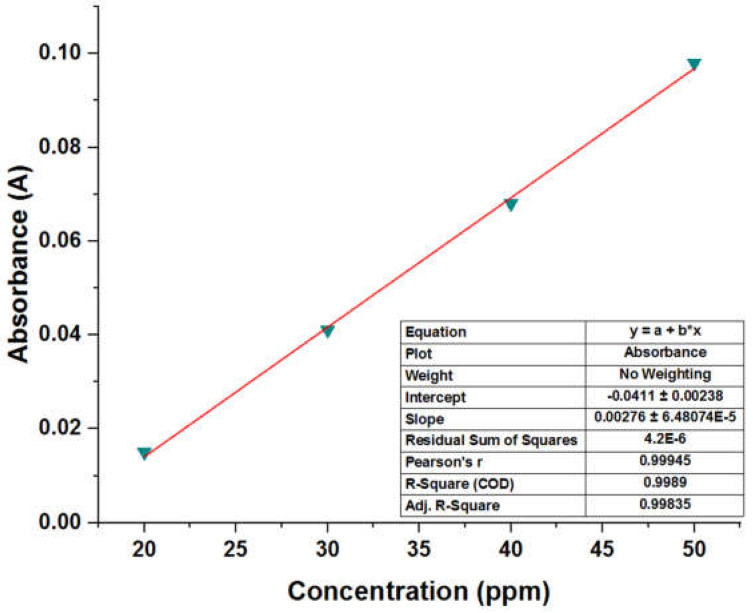
Calibration curve of Millad NX 8000 pure and recovery.

Even more diluted solutions were prepared to carry out the calibration curve, thus taking the concentration points shown in Table 5, since for concentrations below the minimum concentration shown in Table 5, it was found that the behavior of the curve was not linear, and therefore unreliable. An initial sweep was carried out on each of the test samples to determine if the concentration was outside the points of the curve (>50 ppm). For those values that exceeded the curve’s range, dilutions were performed using known integer dilution factors until the sample’s scan matched or closely resembled that of the standard. Once this was achieved, the obtained absorbance value was subjected to the established model, namely the calibration curve (Figure 7), to determine the concentration of the diluted sample. The product of the dilution factor and the concentration of the dilution was then calculated to obtain the actual concentration of the original sample (undiluted sample). For the sample containing Millad NX 8000, the λ_max_ was determined to be 284 nm, which represented a measurement error of 0.7%.

The parameterization results of the points obtained show that the linear equation is y = 0.0028x − 0.0411, with a coefficient of determination (R^2^) = 0.9985 and a correlation coefficient of 0.99945. The results of the linearity test measurements show that the absorbance value and the correlation coefficient are valid and reliable in determining the concentrations. The correlation coefficient value obtained in this validation process meets the requirements for validation acceptance (R^2^ > 0.99). These results denote that the curve has an acceptable linearity between concentration and absorbance, complying with Lambert Beer’s law, which states that the higher the concentration, the greater the absorbance produced [31].

### 2.4. Spectral Analysis

**Figure 8:**
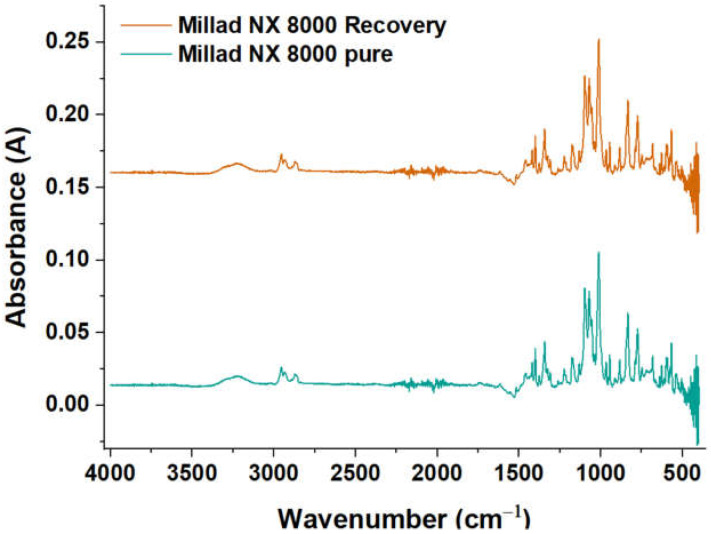
Infrared spectrum for Millad NX 8000 pure and recovered.

The FTIR spectra of pure and recovered Millad NX 8000 are found in Figure 8. Comparing the spectra, there are similarities in the peaks of both samples. For these spectra, it is found that the nucleation agents in PP present a characteristic absorption peak around 1073 cm^−1^, which corresponds to the stretching of the C–O bond and the C–O–C bond, which indicates the existence of an acetal [32]. The vibration that produces a set of peaks with high intensity in absorption between 1000 and 1200 cm^−1^, which are due to the vibration coupling of the C–O bond in the acetal, and the stretching vibration of the C–OH bond of the hydroxyl group, were also observed in-plane bending of the C–H benzene ring [33]. In Figure 8, a characteristic absorption peak is observed at 1073 cm^−1^ in both samples. The characteristic peak at 1073 cm^−1^ is used to characterize and quantify the nucleating agent Millad NX 8000 because it represents a specific vibration associated with the functional groups present in this nucleating agent. Each compound has a unique spectral signature that corresponds to the specific molecular vibrations of the chemical bonds present in its structure. In the case of the nucleating agent Millad NX 8000, the vibrational peak at 1073 cm^−1^ has been identified as a distinctive feature of this compound that allows for the specific identification, quantification and the determination of the presence of this nucleating agent in a polymeric material. Using infrared spectroscopy techniques, such as Fourier transform infrared spectroscopy (FTIR), the interaction between infrared light and the nucleating agent molecules can be analyzed.

The spectrum reveals the presence of characteristic bands associated with Millad NX 8000, with an average intensity between 500 and 100 cm^−1^. In addition, strong peaks can be seen at 750 cm^−1^, corresponding to the stretching vibration of the C–H band in the aromatic ring.

#### Application of Recovered Additive in Polypropylene Resins to Verify Their Efficiency

**Figure 9:**
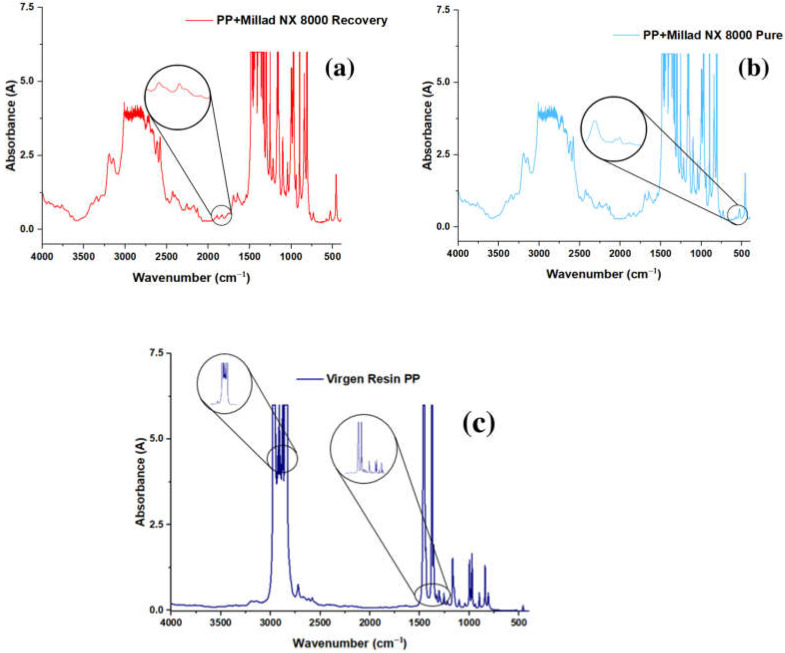
(**a**) PP + infrared spectrum Millad NX 8000 Recovered, (**b**) PP + infrared spectrum Millad NX 8000 pure, (**c**) Infrared spectrum of virgin PP.

The recovered and pure additive incorporation in the virgin PP resin was evaluated using infrared spectroscopy. In Figure 9c, the absence of the peak at 682 cm^−1^ characteristic of the additive, both recovered and pure, is observed in the spectrum of the virgin resin. The said peak can be attributed to the vibration of the C–Hd(mono) bond for aromatic rings. In addition to strong peaks around 3000–3020 cm^−1^, attributed to the C–Ht bonding of the aromatic rings, the peak at 3400 cm^−1^, attributed to the O–Ht vibrational bonding, indicates that the additive has been successfully incorporated into the polymer matrix [34,35]. The presence of the characteristic Millad NX 8000 peaks in the spectrum of the PP with the additive indicates that the additive is still present after processing and has not degraded significantly. In addition, additive-specific characteristic peaks in the spectrum can be used to quantify the amount of additive present in the polymer. The analysis of the additive in the polymer was carried out with concentrations of 1000, 2000 and 4000 ppm.

### 2.5. Thermal Properties

#### 2.5.1. DSC Analysis

**Figure 10:**
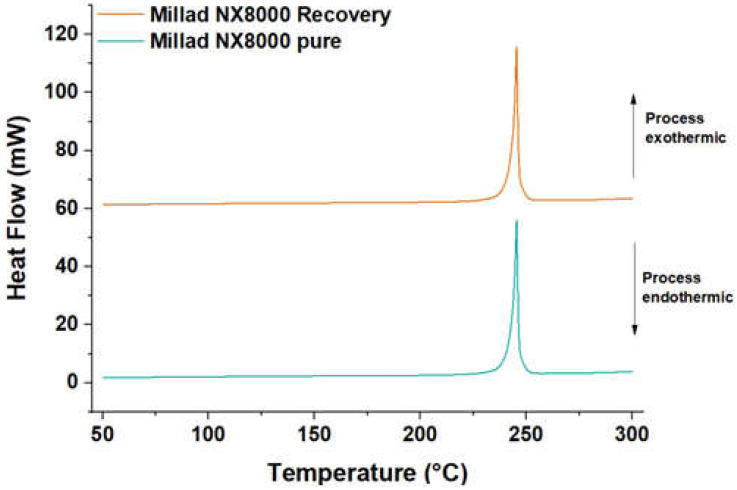
DSC Millad NX 8000 pure and recovered.

Figure 10 illustrates the transformation and melting temperatures of pure and recovered Millad NX 8000, as determined via differential scanning calorimetry (DSC). The specific values for the transformation and melting temperatures of the pure and recovered additives can be found in Table 6. The findings indicate that the pure additive has a transformation temperature of 198.9 °C and a melting temperature of 245.7 °C. Similarly, the recovered additive exhibits a transformation temperature of 199.3 °C and a melting temperature of 245.7 °C. These results suggest that the compound possesses favorable thermal and recrystallization stability, indicating a high degree of purity in the recovered additive. Importantly, the transformation temperatures consistently fall significantly below the melting temperatures. It is worth noting that achieving satisfactory polymer clarity often requires transformation temperatures much lower than the melting point of the fining agent.

Other studies support this information, for example, the case of 1,3:2,4-bis(3,4-dimethylbenzylidene)sorbitol, commonly known as Millad 3988. In this investigation, Millad 3988 showed a transformation temperature of 226 °C and a melting temperature of 273.7 °C. Similar to the above findings, the transformation temperatures were found to be lower than the melting temperatures [35].

#### 2.5.2. TGA Analysis

**Figure 11:**
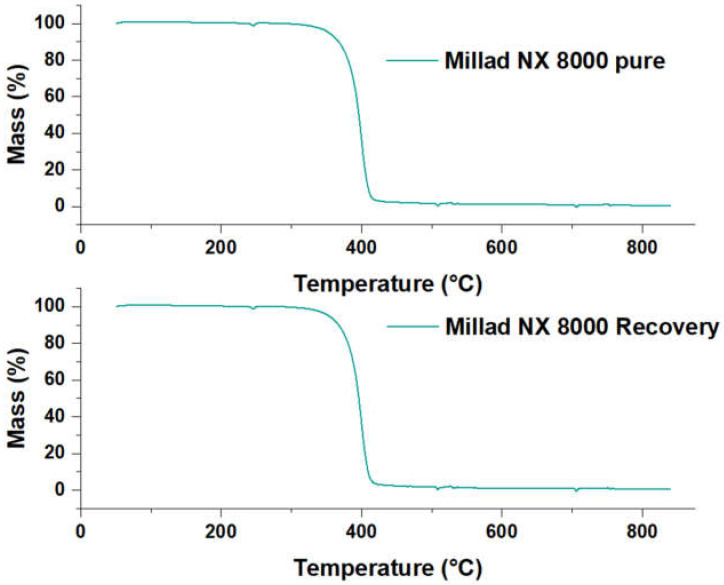
TGA Millad NX 8000 pure and recovered.

Figure 11 shows the TGA results of the recovered pure Millad NX 8000. The graph shows the mass percent as a function of sample temperature for both samples under a nitrogen purge. In the thermal degradation of pure Millad NX 8000, it is observed that the percentage by weight remained stable at 100% until reaching 341 °C, after which the mass of the sample begins to decay. The most relevant percentage weight loss of the sample occurs between 340 °C and 420 °C, after which the degradation continues subtly. What is observed from the recovered Millad NX 8000 is that it follows the same thermal degradation behavior as the pure Millad NX 8000, obeying a first-order transition, since we can see that the material properties change abruptly between 340 and 420 °C. Weight loss during TGA analysis is due to thermal decomposition of the sample. In this case, the Millad NX 8000 shows significant weight loss in the temperature range mentioned, suggesting that the material is susceptible to oxidative degradation in that specific area.

Oxidative degradation occurs when the material is exposed to oxygen and chemical reactions occur that result in the breaking of molecular bonds. This can lead to loss of properties and deterioration of the material. Therefore, it is recommended to perform this analysis in an inert atmosphere.

**Figure 12:**
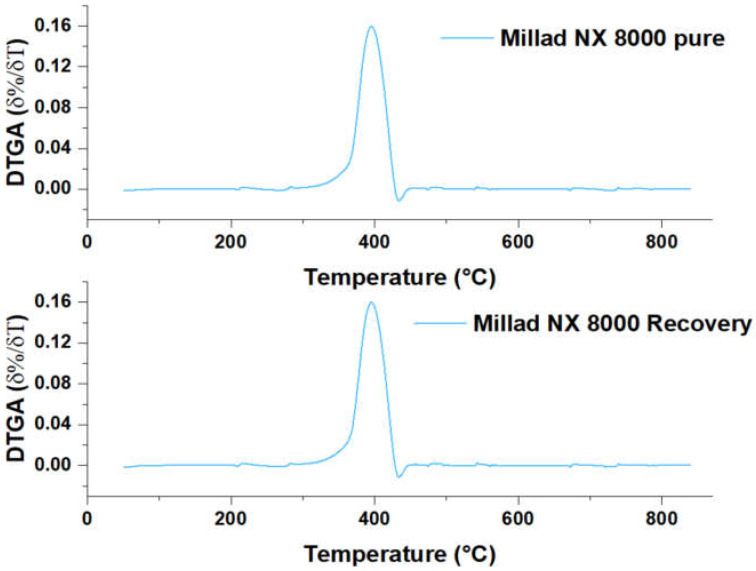
DTGA Millad NX 8000 pure and recovered.

To corroborate the high degree of similarity between the pure and the recovered Millad NX 8000, the pure and recovered DTGA, respectively, were plotted in Figure 12 immediately below the TGA, and here the same maximum value is evident, precisely at a temperature close to that observed in Figure 11 (400 °C), for both derivatives. This indicates the inflection point for pure and reclaimed Millad NX 8000, revealing that the thermal stability of pure and reclaimed Millad NX 8000 is similar.

## 3. Materials and Methods

### 3.1. Sample Preparation

**Figure 13:**
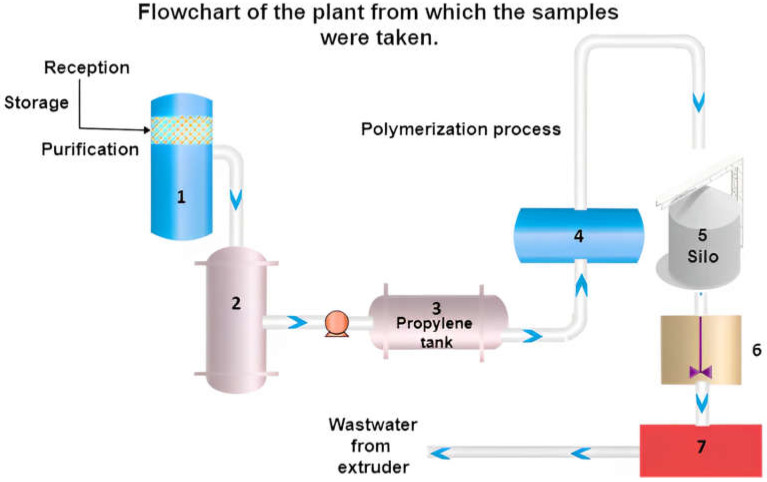
Flow diagram of the plant in which the samples were taken.

Samples were collected at a polypropylene manufacturing plant. Figure 13 shows the stages of the polymer production. The first stage is the reception and purification of the propylene, which is stored in tank one and then proceeded to purify in column 2. In column 2, the elimination of traces of contaminants is conducted; from here, purified propylene is obtained, which is stored in tank 3. In the second stage, propylene is transformed into PP in the polymerization reactor (point 4). The third stage is activation and granulation; in this part of the process, the received virgin resin from the silo (point 5) is mixed with additives (point 6). Extrusion and granulation (pelletization) are carried out at point 7. At this stage, a water flow exits the extruder, which is used to cool and create uniform grains at high temperature. Due to the large number of materials that are processed and the speed of the process, samples were taken every 12 min, with a total of 5 samples obtained. During 50 min, around 30 tons of PP can be processed. Since the process in which contaminants or impurities are removed from the sample takes four hours (desorption), samples were taken from the desorption unit in duplicate every hour.

### 3.2. Addition of the Recovered Additive to the PP Matrix

#### 3.2.1. Preparation of PP Sampling

The PP resin was obtained from a resin without additives (virgin resin) and blended with a mixture of recovered PP and Millad NX 8000 (0.1% by weight). The recovered additive was first mixed with the PP powder in a standard Prodex Henschel 115JSS mixer at a speed of 700 rpm for 7 min at room temperature. Afterward, it was combined with the PP resin. An extruder was then used to produce molten blends formed into films (300 mm diameter with 100 mm thickness) via compression molding in a hot press (CARVER 3895). After granulating the PP solid, recovered granules containing Millad NX 8000 is produced. To make the standard, Millad NX 8000 was diluted in ACN and in a solution with a specific concentration (500 mg/L concentration to obtain 25 mg/L).

#### 3.2.2. Derivatization of the NX 8000 Millad

The derivatization is optimized concerning time and temperature. The optimal time was 30 min, and the temperature was 150 °C. To do this, a standard solution of Millad NX 8000, containing approximately 50 mg L^−1^, was prepared. Briefly, 100 μL of this standard solution, 100 μL of pyridine, and 100 μL of silane reagent (BSTFA: TMCS 99:1) were transferred to a 2 mL vial. To adjust the final volume (2 mL), THF is added. Subsequently, the vial is sealed with a metal cap with a Teflon septum and then placed on a SiC plate. At the end of the experimental stage, the samples are allowed to cool before carrying out the analysis in the chromatograph. Detection and separation of the derivatized Millad NX 8000 were performed using Agilent 6890N/5973N gas chromatography coupled to mass spectrometry (GC/MS) (Agilent Technologies, Palo Alto, CA, USA). The carrier gas was helium, with a constant flow rate of 1.6 mL min^−1^. We worked with a column of activated carbon with fiberglass with dichloromethane. The sample injection system was performed in splitless mode, injecting one μL for all samples. A Lyner with deactivated quartz wool (Skys Deactivation, Bellefonte, PA, USA) was used at the injection port. The separation of the analytes was carried out in the GC oven with an initial temperature of 40 °C (0 min) until reaching 370 °C (2 min) at a ramp of 30 °C min^−1^. The line MS transfer rate remained constant at 300 °C throughout the run. Detection was run in the electron ionization (EI) mode at 70 eV with source and quadruple temperatures of 230 °C and 150 °C after a 3 min solvent delay. Finally, the mass spectra were recorded in scanning mode at 50 to 800 *m*/*z*.

#### 3.2.3. Instrumentation and Spectral Acquisition

FTIR spectra of polypropylene samples were collected in absorbance mode using a Nicolet 6700 FTIR spectrometer in the spectral region of 600–4000 cm^−1^, which were recorded with a resolution of 2 cm^−1^. Samples for FTIR measurements were prepared using filming PP granules, which had a thickness of 300 μm by hot pressing at 400 °C, to cause thermal deterioration and to be able to observe the change in the polymer matrix.

#### 3.2.4. Thermal Properties

Differential scanning calorimetry (DSC) was analyzed on a DSC Q2000 V24.11 Build 124 instrument. Results were obtained under nitrogen and oxygen atmosphere conditions using a 6.0 mg sample. The experimental setup was modified to study how oxidation affected the volatility of the material. Nitrogen gave us a controlled inert environment to study how degradation affects the sample. This procedure was performed under the following condition: isothermal for 5 min, with a 20 °C/min ramp at 350 °C in a nitrogen atmosphere. A change in the exothermic curve was observed due to the oxidation flux.

The measurements to determine the thermal and oxidative stability, in addition to the loss or gain of mass of the polymer, were carried out via the TGA technique, using a Perkin Elmer TGA7 thermobalance from 30 to 700 °C at a speed of 50 mL/min with N_2_. The DTG curve was used to calculate the maximum degradation temperature.

#### 3.2.5. Procedure for the Calibration Curve

A 50 ppm stock solution of Millad NX 8000 was prepared in pure analytical grade toluene as solvent, and an Evolution 60 S UV-Vis spectrophotometer (Thermo Scientific, Waltham, MA, USA) was used for analysis. To prepare different concentrations, aliquots of the standard solutions were transferred into a series of standard 5 mL volumetric flasks, and the volumes were made up of the solvent used. Four 20–50 ppm concentrations of Millad NX 8000 in toluene were prepared for the standard calibration curve. The Millad NX 8000 was rated at 286 nm.

##### Validation of Analytical Method via UV-Vis

*Linearity*

Solutions of neat and recovered Millad NX 8000 were prepared in toluene. All solutions were scanned from 190 to 800 nm, and changes in absorbance at the respective wavelengths were verified. To establish the linearity of the proposed method, four separate solutions of Millad NX 8000 with toluene were prepared from the stock solutions and analyzed. A least squares regression analysis was performed for the data obtained (Table 5).

## 4. Conclusions

In summary, this study not only provides extraction and recovery procedures for Millad NX 8000 but also highlights the benefits associated with recovering nucleating agents, including the additive examined in this study, from industrial wastewater. Previous research has confirmed the migration of such substances to ecosystems and food, posing specific risks. The findings of this study demonstrate that over 96% of Millad NX 8000 can be recovered with high purity. Consequently, incorporating it into the PP matrix can prove highly efficient for intended applications. The thermal and thermo-oxidative stability of the recovered additive closely resembles that of the original additive, as evidenced by the identical FTIR fingerprint, indicating its high purity. Thus, if the recovery process can be scaled up while maintaining these high levels of purity, as achieved in this research, the methodology can be deemed valid, effective, and applicable in the industrial sector. By embracing this approach, we position ourselves at the forefront of supporting the implementation of the circular economy plan within the industry.

## Figures and Tables

**Table 1:** Data obtained for extraction/recovery of Millad NX 8000 in GC-MS with a column of activated carbon plus glass fiber with DCM.

GC-MS with Activated Carbon Column + Fiberglass with DCM										
***Repeatability***										
**Theoretical**	**STDA**	**Analyst 1**	**Analyst 1**	**Analyst 1**	**Analyst 1**	**Average**	**Deviation**	**RSD**	**Error**	**%Recovery**
0	1	0	0	0	0	0	0	0		
500	2	457	468	463	472	465	6.48	1.393	7	93
1000	3	961	975	968	955	964.75	8.655	0.897	3.525	96.47
2000	5	1938	1957	1963	1931	1947.25	15.196	0.78	26.375	97.36
5000	7	4865	4677	4721	4638	4725.25	99.144	2.098	5.495	94.5
***Reproducibility***										
**Theoretical**	**STDA**	**Analyst 1**	**Analyst 2**	**Analyst 3**	**Analyst 4**	**Average**	**Deviation**	**RSD**	**Error**	**%Recovery**
0	1	0	0	0	0	0	0	0		
500	2	469	472	450	447	459.5	12.819	2.789	8.1	91.9
1000	3	947	969	986	942	961	20.379	2.121	3.9	96.1
2000	5	1952	1964	1948	1944	1952	8.641	0.443	2.4	97.6
5000	7	4869	4782	4928	4933	4878	70.289	1.441	2.44	97.56

**Table 2:** ANOVA analysis for patterns and samples using the Tukey method with 95% confidence.

**Repeatability for Standard by GC-MS**				**Reproducibility of the Standard by GC-MS**			
**Factor**	**N**	**Average**	**Group**	**Factor**	**N**	**Average**	**Group**
Analyst 1 (1)	4	2055	A	Analyst 3	4	2078	A
Analyst 1 (3)	4	2029	A	Analyst 4	4	2067	A
Analyst 1 (2)	4	2019	A	Analyst 1	4	2059	A
Analyst 1 (4)	4	1999	A	Analyst 2	4	2047	A
**Repeatability of Patterns with Activated Carbon Column in GC-MS**				**Reproducibility of Patterns with activated Carbon Column in GC-MS**			
**Factor**	**N**	**Average**	**Group**	**Factor**	**N**	**Average**	**Group**
Analyst 1 (4)	4	2055	A	Analyst 3	4	2078	A
Analyst 1 (2)	4	2029	A	Analyst 4	4	2067	A
Analyst 1 (1)	4	2019	A	Analyst 1	4	2059	A
Analyst 1 (3)	4	1999	A	Analyst 2	4	2047	A

**Table 3:** Data obtained for the Millad NX 8000 calibration curve in GC-MS with DCM.

Calibration Curve of the Millad NX 8000 in GC-MS with DCM									
***Repeatability***									
**Theoretical**	**STDA**	**Analyst 1**	**Analyst 1**	**Analyst 1**	**Analyst 1**	**Average**	**Deviation**	**RSD**	**Error**
0	1	0	0	0	0	0	0	0	
500	2	485	492	495	488	490	4.4	0.9	2
1000	3	972	986	991	991	985	9.0	0.9	1.5
2000	5	1978	1991	1986	1988	1986	5.6	0.3	0.7
5000	7	4978	4969	4989	4992	4982	10.6	0.2	0.4
***Reproducibility***									
**Theoretical**	**STDA**	**Analyst 1**	**Analyst 2**	**Analyst 3**	**Analyst 4**	**Average**	**Deviation**	**RSD**	**Error**
0	1	0	0	0	0	0	0	0	
500	2	487	496	476	477	484	9.416	1.945	3.2
1000	3	968	955	986	982	972.75	14.127	1.452	2.725
2000	5	1990	1958	1973	1957	1969.5	15.503	0.787	1.525
5000	7	4997	4957	4961	4997	4978	22	0.441	0.44

**Table 4:** Tabulation of data for sample reproducibility.

Analysis of Final Samples with Activated Carbon Column + Fiberglass with DCM											
*Reproducibility*											
Day	Sample	Analyst 1	Analyst 2	Analyst 3	Analyst 4	Average	Deviation	RSD	Error	Recovered	%Recovery
1	1	4254	4385	4568	4449	4414	130.871	2.964	65.435	4257	96.443
2	2	3875	3789	3526	3911	3775.25	173.87	4.605	86.935	3524	93.344
3	3	1542	1436	1342	1402	1430.5	838.788	5.863	41.939	1324	92.555
4	4	965	878	978	911	933	46.754	5.011	23.377	901	96.57
5	5	2145	2218	2086	2115	2141	56.703	2.648	28.351	2000	93.414
6	6	1578	1531	1496	1511	1529	35.674	2.333	17.837	1452	94.964
7	7	1978	2175	2088	1952	2048.25	103.028	5.03	51.514	1955	95.447
8	8	875	792	854	863	846	370.135	4.375	18.506	811	95.863
9	9	1478	1542	1575	1437	1508	621.449	4.121	31.072	1425	94.496
10	10	3478	3574	3485	3314	3462.75	108.367	3.129	54.183	3375	97.465
11	11	2756	2811	2901	2754	2805.5	68.927	24.568	34.463	2578	91.89
12	12	1900	1921	1945	1805	1892.75	613.208	32.397	30.66	1785	94.307
13	13	758	777	765	738	759.5	16.34	2.151	8.17	743	97.827
14	14	935	943	924	911	928.25	13.889	1.496	6.944	899	96.848
15	15	1758	1792	1732	1675	1739.25	49.378	2.839	24.689	1608	92.453
16	16	785	793	777	713	767	365.877	4.77	18.294	718	93.611
17	17	475	497	481	445	474.5	217.485	4.583	10.874	457	96.311
18	18	511	542	552	499	526	25.073	4.766	12.536	508	96.577
19	19	348	327	337	311	330.75	15.713	4.75	7.856	324	97.959
20	20	311	308	316	335	317.5	12.124	3.818	6.062	299	94.173
21	21	268	285	293	299	286.25	13.45	4.698	6.725	264	92.227
22	22	467	477	465	445	463.5	134.039	2.891	6.702	435	93.851
23	23	526	551	524	511	528	167.132	3.165	8.356	508	96.212
24	24	611	634	611	578	608.5	230.434	3.786	11.522	586	96.302
25	25	642	689	701	721	688.25	335.397	4.873	16.769	654	95.023
26	26	719	754	716	688	719.25	270.477	3.760	13.524	675	93.847
27	27	366	352	342	327	346.75	164.392	4.74	8.219	337	97.188
28	28	485	511	499	542	509.25	242.813	4.768	12.14	491	96.416
29	29	290	281	291	278	285	64.807	2.273	3.24	275	96.491
30	30	421	415	402	384	405.5	163.808	4.039	8.19	383	94.451

**Table 5:** Results of the linearity test of the UV-Vis spectrophotometry method.

Concentration (ppm)	Absorbance
20	0.015
30	0.041
40	0.068
50	0.098

**Table 6:** Predicted critical temperatures of sorbitol clarifiers.

Clarifier	*T*_t_ (°C)	*T*_m_ (°C)
Millad NX 8000 pure	198.9	245.7
Millad NX 8000 recovery	199.3	245.7

*T*_t_—Transformation temperature, *T*_m_—Melting temperature determined via DSC.

## Data Availability

The data presented in this study are available on request from the corresponding author.

## Sample Availability

Samples of the compounds are not available from the authors.
